# Incomplete faces are completed using a more average face

**DOI:** 10.1186/s41235-022-00429-y

**Published:** 2022-08-19

**Authors:** Robin S. S. Kramer, Alex L. Jones

**Affiliations:** 1 School of Psychology, University of Lincoln, Brayford Pool, Lincoln, LN6 7TS UK; 2 School of Psychology, Swansea University, Swansea, SA2 8PP UK

**Keywords:** Facial attractiveness, Facial typicality, Positivity bias, Upper- and lower-face, Face morph, Face average

## Abstract

Facial first impressions are known to influence how we behave towards others. As a result of the COVID-19 pandemic, we often view incomplete faces due to the commonplace wearing of face masks. Previous research has shown that perceptions of attractiveness are often increased due to these coverings, with initial evidence suggesting that this may be caused by viewers using a mental representation of the average face to complete any missing information. Here, we directly address this hypothesis by presenting participants with incomplete faces (either the lower or upper half removed) and asking them to decide how they thought the actual, full face looked. Participants were able to manipulate the missing half of the face onscreen by increasing or decreasing the averageness of its shape. Our results demonstrated that participants did not select the original versions of the faces but instead chose more average versions when manipulating both the lower and upper face. Further, the typicality of the original image influenced responses, with less typical faces (in comparison with more typical ones) being completed using an even more average version of the missing half of the faces. Taken together, these findings provide the first direct evidence that people utilise an average/typical internal representation when inferring information about incomplete faces. This result has theoretical importance in terms of visual perception, as well as real-world relevance in a time where face masks are commonplace due to the COVID-19 pandemic.

**Supplementary Information:**

The online version contains supplementary material available at 10.1186/s41235-022-00429-y.

## Introduction

Due to the COVID-19 pandemic and the everyday use of face masks (Rab et al., 2020), we have now become accustomed to forming facial first impressions based on incomplete information. Recent studies have begun to address how the wearing of face masks may have impacted our rapid and automatic judgements of others (Olson & Marshuetz, 2005; Ritchie et al., 2017; Willis & Todorov, 2006), given that our initial impressions of strangers can have significant implications for how we behave towards them. The ‘halo effect’ (Dion et al., 1972), for example, describes how socially desirable traits are indiscriminately applied to attractive people, who in turn, may benefit from receiving more help (Benson et al., 1976), getting more lenient sentences in court (Erian et al., 1998), and earning higher wages (Pfeifer, 2012).

A growing body of research has focussed on the nature of attractiveness perceptions when faces are either partly obscured by masks or entirely visible to the viewer. Prior to the pandemic, Miyazaki and Kawahara (2016) showed that faces originally categorised as middle- or high-attractiveness suffered a decrease in ratings when presented with face masks. The authors argued that this pattern of results supported the ‘sanitary-mask effect’, whereby the presence of masks primed perceptions of illness/poor health. In contrast, when the lower face was occluded by a notebook instead, high-attractiveness faces were rated lower, while low-attractiveness faces were rated higher. Here, the underlying mechanism was argued to be the result of the occlusion itself. Covering the lower part of unattractive faces caused an increase in ratings (e.g., by hiding signs of low symmetry and rough skin) while the same occlusion caused a decrease in ratings for attractive faces (e.g., by hiding signs of high symmetry and smooth skin).

Subsequent research has taken place since the onset of the pandemic, where our exposure to face masks has presumably negated the above-mentioned negative primes associated with mask-wearing. Indeed, studies have tended to show an overall increase in attractiveness perceptions with the presence of face masks, or at least, no overall decrease in ratings. For instance, Hies and Lewis (2022) found that face masks increased ratings of attractiveness, and this was equally true for both unattractive and attractive faces. Further, when faces were considered without categorising into low and high groups, no overall differences were found when rating faces with versus without face masks (Bennetts et al., 2022; Guo et al., 2022).

Interestingly, many researchers have also identified different patterns of results for low- and high-attractiveness faces, perhaps supporting the mechanism described above (Miyazaki & Kawahara, 2016). For example, Patel et al. (2020) found that faces when presented with masks were rated as more attractive in general, although this was particularly true for the most unattractive faces (which saw the largest increase). In addition, Kamatani et al. (2021) showed that high-attractiveness faces decreased in their ratings when presented with masks, while low-attractiveness faces showed an increase in their ratings. Finally, Pazhoohi and Kingstone (2022) also found that unattractive faces were rated as more attractive when presented with masks, although attractive faces saw no change in how they were perceived. This pattern was also present in a study by Oldmeadow and Koch (2021), although these researchers initially categorised their stimuli based upon trustworthiness rather than attractiveness: only low-trustworthiness faces increased in attractiveness ratings when presented wearing masks, which is consistent with previous research that has established a strong correlation between facial trustworthiness and attractiveness perceptions (e.g., Oosterhof & Todorov, 2008).

How might we account for these varying patterns of results? As Pazhoohi and Kingstone (2022) note, it may be that cultural differences represent one cause. For instance, both stimuli and participants were Japanese in some studies (Kamatani et al., 2021; Miyazaki & Kawahara, 2016) and Western in others (Pazhoohi & Kingstone, 2022). In addition, due to the variation in stimuli across studies, the levels of attractiveness of the faces used may have played an important role in the results. For example, if one study found that face masks lowered the ratings of high-attractiveness faces while another did not, this might be explained by differences in how attractive this category of faces was initially perceived to be across studies.

There are other potential issues that might account for some of these discrepancies that are more statistical in nature. First, several studies categorised their stimuli (low- and high-attractiveness, for instance) when their original attractiveness ratings were continuous (e.g., Patel et al., 2020; Pazhoohi & Kingstone, 2022). Dividing such a distribution into arbitrary categories can misrepresent the true pattern of data, increase the risk of false positives, and lower the power of the experiment to measure the true correlation of attractiveness with and without face masks (Altman & Royston, 2006; MacCallum et al., 2002; McClelland et al., 2015). Second, the pattern described earlier suggesting high-attractiveness faces are expected to decrease in their ratings when presented with masks while low-attractiveness faces should demonstrate the opposite pattern (Kamatani et al., 2021; Miyazaki & Kawahara, 2016) could simply be a result of regression towards the mean. Attractiveness with and without masks will be imperfectly correlated, with random factors playing a roll. Such statistical problems have also been highlighted in other fields including metacognition (for an in-depth discussion of these issues, see Kramer et al., 2022).

Given the current lack of agreement in the attractiveness predictions we should make when occluding the lower face, we have instead chosen to focus on the underlying mechanism in the current study since this may prove more informative. To this end, Orghian and Hidalgo (2020) carried out several experiments in which their overall findings demonstrated that incomplete facial photographs (e.g., presenting a vertical slice of the image, or the whole image but with missing pixels) were rated as more attractive than the original images—termed a ‘positivity bias’. When participants are instructed to judge the attractiveness of incomplete faces, they are forced to use prior knowledge of faces more generally (i.e., structure and features). Further, they may rely on their representation of a prototypical face, and use this in order to generate new, complete representations. This prototypical representation is considered to be the statistical average of all seen faces, and provides the basis for a neural ‘face space’, where other faces can be encoded relative to this prototype (Valentine, 1991). Indeed, evidence suggests that the formation of face prototypes is both rapid and implicit (de Fockert & Wolfenstein, 2009; Kramer et al., 2015; Neumann et al., 2013; Or & Wilson, 2013), and may even take place in infancy (de Haan et al., 2001).

If incomplete faces are completed using a facial average or prototype (or, at least, assumptions based on this representation) then we should expect an increase in attractiveness ratings for incomplete images (due to the highly attractive nature of averages; Langlois & Roggman, 1990). Further, we might predict that this increase is dependent on the original typicality of the face (i.e., its proximity or similarity to the prototype)—for an already typical face, occlusions or missing information should result in less of an attractiveness increase. Indeed, evidence appears to support this pattern of results (Experiment 6; Orghian & Hidalgo, 2020).

Here, we begin to investigate the mechanisms underlying the positivity bias. By providing only half of an original image, while allowing participants to manipulate the other half to depict a more or less typical version, we target the participant’s internal representation itself. Tasked with selecting the version that most closely resembles how the complete face actually looks (according to the participant), we are able to consider directly the question of whether incomplete faces are completed using the average face. Further, through the use of perceived and measured typicality of the original faces, we can explore whether less typical faces (in comparison with more typical ones) will be completed by participants using an even more typical version of the missing part of the face.

## Methods

### Participants

A sample of 110 volunteers (80 women; age *M* = 21.5 years, SD = 6.2 years; 93% self- reported ethnicity as White) gave informed consent before participating in the experiment (in person) and were provided with both a written and verbal debriefing upon completion. Participants were recruited by word of mouth (e.g., through asking friends and family), as well as through the university’s SONA participant panel. This experiment was approved by the university’s ethics committee and was carried out in accordance with the provisions of the World Medical Association Declaration of Helsinki.

An a priori power analysis was conducted using G*Power 3.1 (Faul et al., 2007), based on a medium effect size (Cohen’s *d* = 0.5) when comparing participants’ average responses (morph percentage chosen from the sequence; see below) to a value of 0% (the original, unaltered image). To achieve 80% power at an alpha of 0.05, a total sample size of 34 was required. However, given that participants were assigned to one of two conditions (manipulating either the upper or lower half of the face), we aimed for at least 34 participants per condition.

### Stimuli

From a larger set of facial photographs of university students, previously collected and featured in other studies (e.g., Jones et al., 2012), we selected all White models aged between 18 and 30 years old. These 240 identities (137 women; age *M* = 20.8 years, SD = 2.0 years) were photographed with a neutral expression, at a fixed distance to the camera, and with their hair pulled back from their faces as much as possible, with cosmetics and jewellery removed.

First, using custom MATLAB software (The Mathworks, Natick, MA), these 240 images were rotated so that both pupils were aligned to the same transverse plane. Next, we used JPsychoMorph software (Tiddeman et al., 2001) to delineate the images by manually applying 156 anatomical landmarks across the internal features and outline of the face (excluding the ears, neck, and the top of the head). Finally, these shapes, along with their associated images, were then used to create two averages—one derived from all the male faces and one from all the female faces.

From the set of 240 identities, 60 of these (30 women) were randomly chosen to produce our experimental stimuli. For each face, we used the above shapes to produce two subsets of landmarks, comprising either the upper half (85 points covering the upper cheeks, upper part of the nose, eyes, eyebrows, and hairline) or lower half of the face (71 points covering the lower cheeks, lower part of the nose, mouth, and jawline). In the stage described below, where morphing continua were created, we completed the process twice—once using only the landmarks from the upper half of the face, and once using only the lower face landmarks. In this way, we were able to produce two sequences of images for each identity, allowing participants to manipulate the appearance of only the upper or lower half of the face while the other half remained unchanged. (In reality, there were very minor changes to the half of the face that was not landmarked due to the software altering that half to form a seamless join with the landmarked half.)

**Fig. 1:**
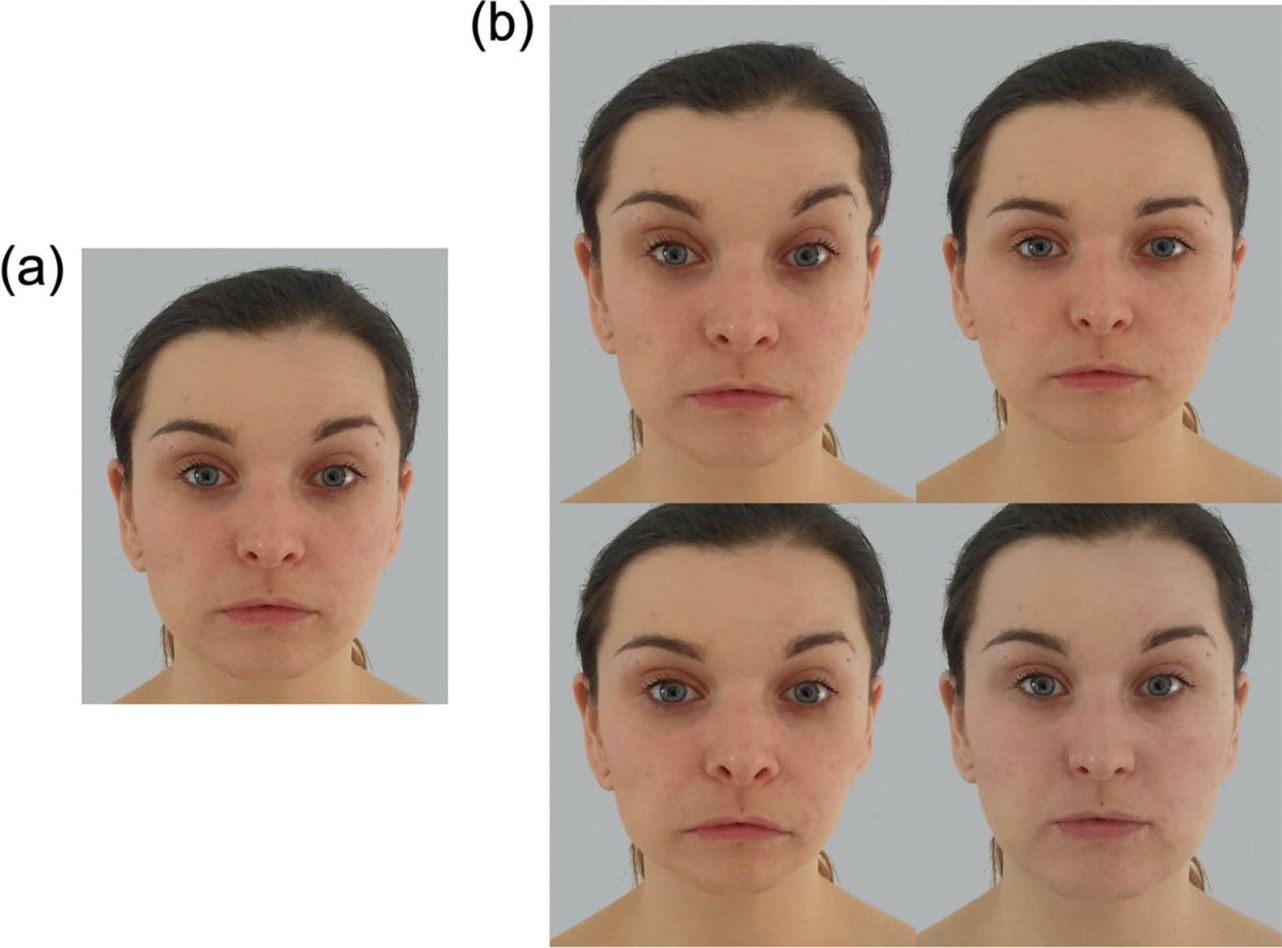
An example identity, morphed to appear more or less average in appearance. **a** The original, unaltered image. **b** Image sequences featured shape changes to only the upper (top row) or lower half of the face (bottom row). Images depict − 50% (left) and +50% morphs (right), where the original image and average were located at 0% and 100%, respectively. This woman did not appear in the experiment itself, and has given permission for her image and morphs to be reproduced here

Using JPsychoMorph, for each face (and utilising the upper and lower landmarks separately), we conceptualised a morphing continuum with the original image located at 0%, and placing the same-sex average image at 100%. We then generated a sequence of 21 images, where the original was morphed between − 50% and + 50% along this continuum in increments of 5% (see Fig. 1). Images below 0% represented caricatures in the sense that they emphasised the face’s distinctiveness from the average (e.g., Itz et al., 2016; McIntyre et al., 2013), while those above 0% are often referred to as anti-caricatures in that the face becomes less distinctive and more average in appearance (e.g., Nishimura et al., 2010). In these sequences, the 0% image represented the unaltered original. It is important to note that we chose to transform only the shape of the face (rather than also including the colour and texture) in order to avoid unnatural smoothing of the skin for anti-caricatures, and unrealistic emphasis of facial blemishes for caricatures.

Therefore, for each face, the above process resulted in two sequences of 21 images. In these, either the upper half of the face morphed to appear more or less average in its shape while the lower half remained unchanged, or the lower half morphed while the upper half remained unchanged. Finally, all images were cropped to include only the head and the top of the shoulders, with the left and right zygions anchored within the frame across images within each sequence in order to produce a smooth change when manipulated by participants. All images were resized to measure 6.1 cm in width and approximately 7.8 cm in height, with heights varying slightly across identities to accommodate different head shapes and changes.

In order to create our target images (i.e., those presented onscreen with only half of the face visible, giving participants limited information about the person’s appearance), we replaced either the upper or lower half of the original image with a white rectangle. This image was also less tightly cropped so as contain more background space around the head. As a result, participants would not be able to match the two images directly when searching for the original within the sequence. This would have otherwise been a potential strategy because, while the unlandmarked half of the *face* remained unchanged across the image sequence, that half of the *image* showed some minor variation as it accommodated the changes in the other half of the face in terms of height, etc. (see above). Therefore, any slight changes in terms of framing within the image might have provided cues to which image in the sequence was the original (0%). By providing the target as a different version of the image in terms of cropping, this was no longer possible.

#### Image ratings

Before the main task presented here, we collected both typicality and attractiveness ratings of the 60 original images from 160 volunteers (100 women; age *M* = 31.0 years, SD = 15.2 years; 89% self- reported ethnicity as White). The data from two additional participants were excluded because the same rating was given for every image. Participants were recruited by word of mouth (e.g., through asking friends and family). There was no overlap between this sample and the participants recruited for the main experiment.

The task was completed online using the Gorilla experiment builder (Anwyl-Irvine et al., 2020). After consent was obtained, participants provided demographic information (age, gender, and ethnicity). On each of the 60 trials, participants were presented with one original image (with order randomised for each participant) and asked to rate either the typicality (*n* = 76) or attractiveness (*n* = 84) of the face using a slider appearing below the image. Participants rated all faces for only one of these traits, with this trait selection counterbalanced across participants. In line with previous research (Orghian & Hidalgo, 2020), for typicality, participants were asked “How much does this face deviate from a typical face?” with responses provided on a scale from 0 (does not deviate at all) to 100 (deviates very much). For attractiveness, participants were asked “How attractive is this face?” with responses provided on a scale from 0 (very unattractive) to 100 (very attractive). Responses were provided using the computer’s mouse, and no time limits were imposed.

#### Geometric typicality measurements

In addition to collecting ratings of perceived typicality, we used geometric morphometric techniques to measure facial shape typicality. First, we aligned the full-face landmarks for each of the 60 original, unaltered faces to their same-sex average shapes (i.e., female faces were aligned to the female shape average) using a Procrustes fit. Next, treating each face shape as a vector, we computed the Euclidean distance between each Procrustes-aligned face and its same-sex average. Therefore, greater values represented a facial shape that was further away from the average. However, where this variable was used in analyses, it was reverse scored such that higher scores reflected a more typical facial shape. This metric has previously been used in several examinations of facial shape and social perceptions (Holzleitner et al., 2019; Jones & Jaeger, 2019).

### Procedure

The experiment was completed in person using custom MATLAB software to carry out the task. After consent was obtained, participants provided demographic information (age, gender, and ethnicity).

First, participants were instructed both verbally and onscreen regarding the goal of the task—on each trial, they should “change the face to indicate how you think the person actually looks.” For each of the 60 trials that followed (with order randomised for each participant), a target image was presented to the left of the screen (3.9 cm in width and approximately 4.7 cm in height). This was the original, unaltered image of the identity but with only half of the face visible. Whether the upper or lower half of the face was shown depended upon experimental condition, with this counterbalanced across participants. As a result, participants only completed trials for ‘upper half only’ or ‘lower half only’, which prevented them from seeing the original upper half of an identity’s face earlier in the experiment and then later being asked to select this half within the sequence, for example.

**Fig. 2:**
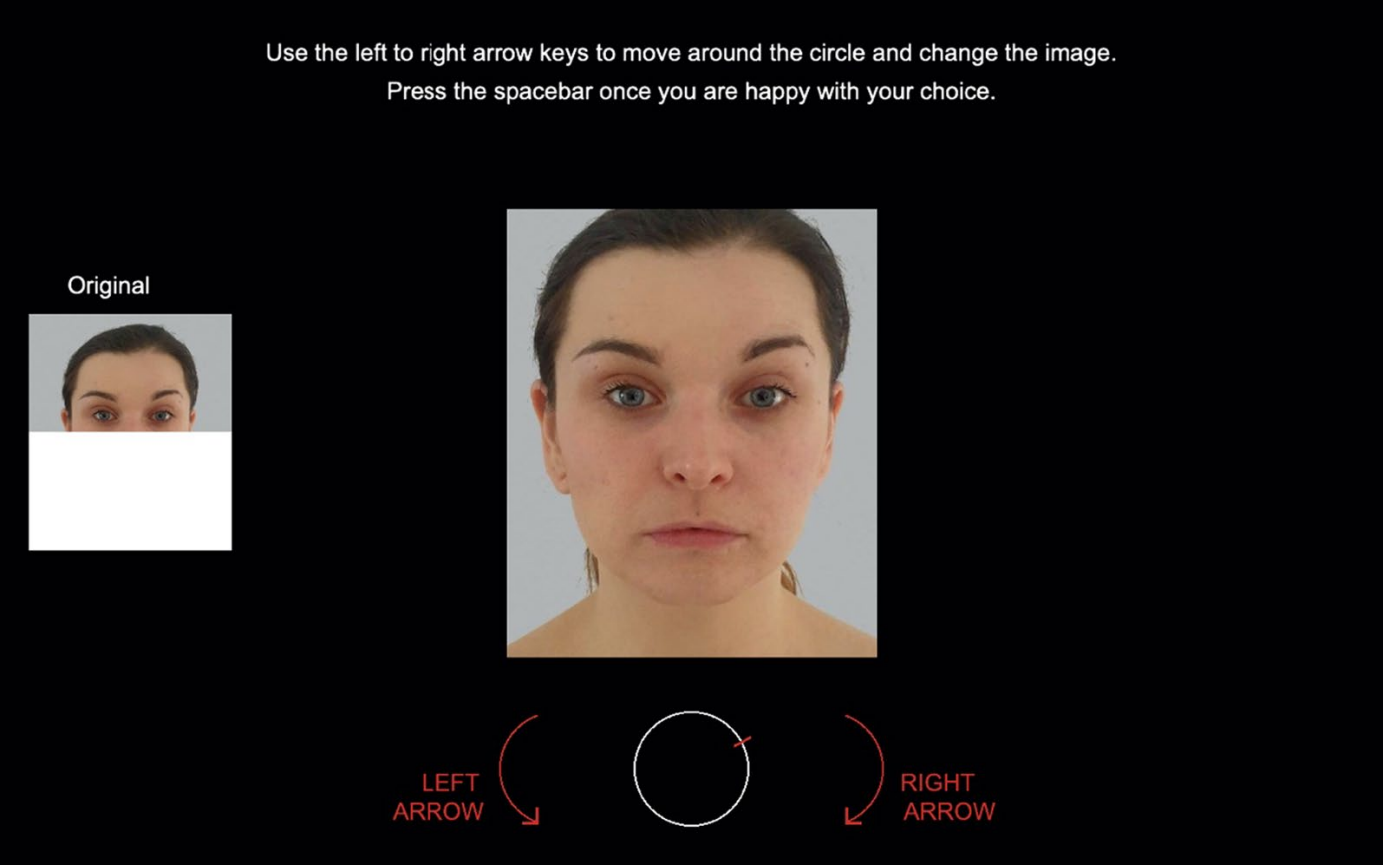
The display presented to participants. In this example, participants are shown only the upper half of the face (left) and asked to manipulate the central image (by cycling through the sequence) in order to select the version corresponding to how they think the person actually looks. Here, the central image depicts the original (0%) image of this identity

The image manipulation task itself followed the design used in previous work (Jones et al., 2014). In the centre of the screen on each trial, a random image from the identity’s morph sequence (see above) was presented. (The sequence represented changes to either the upper or lower half of the face, depending upon the condition to which the participant had been assigned.) Underneath the face was a white circle, with a red bar over a random point of the circumference (see Fig. 2). The bar could be moved around the circle using the left and right arrow keys. Each position of the bar corresponded to an image in the identity’s sequence, and movement of the bar altered the image accordingly. One full cycle caused the image to move smoothly through the complete sequence and back again (− 50% to + 50% to − 50%). For each trial, the starting position of the bar, and the image corresponding to its position, were randomised.

Participants moved through the sequence of an identity with the arrow keys, and pressed the spacebar once they felt they had reached the image which represented how the person actually looked. No time limits were imposed upon responses. The morph percentage corresponding to the selected image was then recorded as the participant’s response, although this value was not seen by participants.

## Results

### Do participants select the original image?

**Fig. 3:**
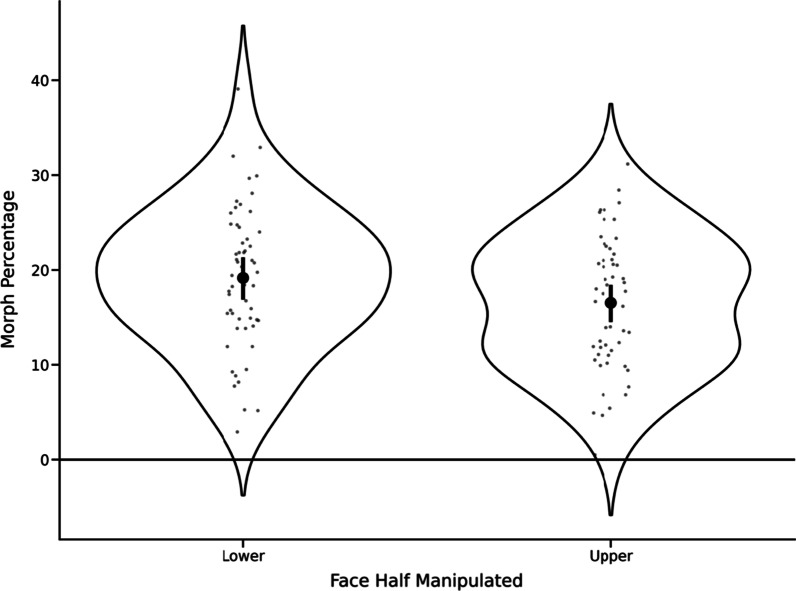
Violin plots illustrating the distributions of the morph percentages for both experimental conditions. Black dots and bars represent the means and 95% confidence intervals, respectively. Grey dots represent the individual participant means. The horizontal line represents a morph percentage of zero, i.e., the original, unaltered version of the face

For each of the 56 participants who were tasked with manipulating the lower face (while the upper face remained constant) so that the resulting images represented how they thought the identities actually looked, we first averaged their responses (morph percentage) across trials. Next, we compared these averages to a value of zero using a one-sample *t*-test. Any statistically significant difference would reflect a systematic pattern of behaviour whereby participants selected versions of the faces that deviated from the original, unaltered image. We found that participants chose images that had been morphed towards the average and away from the original version: *M* = 19.16%, SD = 7.46%; *t*(55) = 19.21, *p* < 0.001, Cohen’s *d* = 2.57 (see Fig. 3).

We then repeated this same analysis for the 54 participants who were tasked with manipulating the upper face (while the lower face remained constant). Again, we found that participants chose images that had been morphed towards the average and away from the original version: *M* = 16.53%, SD = 7.04%; *t*(53) = 17.26, *p* < 0.001, Cohen’s *d* = 2.35 (see Fig. 3).

### Typicality measures

We first averaged the perceived atypicality ratings across participants and reverse scored them, resulting in a measure of perceived typicality for a given face. This process was repeated for the attractiveness ratings (although these ratings were not reversed). We then computed the zero-order correlations between attractiveness, perceived typicality, and measured typicality (generated using the face shapes themselves; see above). Perceived typicality showed a strong relationship with attractiveness, *r*(58) = 0.72, *p* < 0.001, while measured typicality showed a weaker and non-significant positive relationship with attractiveness, *r*(58) = 0.10, *p* = 0.457. The two measures of typicality showed a small, non-significant association with each other, *r*(58) = 0.19, *p* = 0.139.

Given the strong and expected association between perceived typicality and attractiveness found here and in previous work (e.g., Ryali et al., 2020), we chose to focus on typicality only when predicting participants’ morph percentage responses. This decision reflected two considerations. First, by including two strongly correlated predictors in regression analyses, resulting models can suffer from issues with multicollinearity. Second, our argument, based on previous evidence (Orghian & Hidalgo, 2020), is that participants completed faces with missing information using the average or typical face. Therefore, the typicality of the face, and not its attractiveness, is the trait of relevance here.

### Regression analytic strategy

To draw inferences from our experimental data (see Additional file 1), we used a multilevel beta regression to predict the morph percentage selected on each trial by each participant, from both perceived and measured typicality, while accounting for participant- and stimulus-level random effects. Therefore, we had fixed effects for both typicality measures, but used a specific implementation for our random effects—while participants had random intercepts, estimating the average morph percentage for each participant, stimuli had a random slope only for the effect of measured typicality. Consider that measured typicality is computed from the facial landmarks that are also used for transformation of the stimuli. As such, measured typicality is directly tied to the transformation, such that individual steps of the transform used already depend on the distance from an individual face to the average shape. For example, for faces close to the average, the transforms will have smaller steps. A negative association between measured typicality and transform choice is then somewhat deterministic, and a random intercept—the average transform choice for a stimulus—does not correct for that. However, a random slope for measured typicality allows a face-specific effect of measured typicality; such that faces scoring high on measured typicality have a specific slope for their responses. This parameterisation may help to model this aspect of our design.

To estimate this model, we used Bayesian methods, which can flexibly estimate the parameters of non-standard models. Models were estimated using the Bambi (Capretto et al., 2022) and PyMC3 (Salvatier et al., 2016) packages in Python. Before fitting the model, both predictors were *z*-score transformed, and the dependent variable (morph percentage) was scaled to the interval (0, 1) using the method suggested by Smithson and Verkuilen (2006). Thus, a response where the participant selected the original, unaltered face was represented by a value of 0.50 after the transform. Beta regression uses the logit-link function (akin to logistic regression), with coefficients representing log-odds.

Bayesian inference requires the specification of a prior distribution over estimated parameters. We fitted two kinds of models where the prior distribution for both perceived and measured typicality differed. First, we used an informed normal distribution for the coefficients centred at − 0.05 and with a standard deviation of 0.10. On the odds scale, this represented our hypothesis that a negative effect was most likely (such that increasing typicality would lead to lower morph percentage choices), with odds of around 0.90, but that a positive effect could still occur with around 15% probability. A second version of the model was fitted with uninformative, very weak priors that had little influence on the data, to examine how much the estimates of the coefficients changed. For the intercept term, a weak normal prior centred at zero with a wide standard deviation of 2.5 was used, and for the random effects (offsets from the intercept term for participants, and offsets from the slope of measured typicality for stimuli), a standard normal distribution was used, scaled by a half-normal hyperprior with a standard deviation of 2.5 for the offset standard deviation.

Finally, after the models were fitted, we computed the mean and 95% highest density intervals (on the odds-scale) of the posterior distributions of the typicality coefficients, as well as probability of direction (Makowski et al., 2019)—specifically, that the effect was negative. This value represented the probability of the hypothesis (the effect would be negative), given the data, in contrast to a null-hypothesis significance test (the probability of observing data as extreme or more, given no effect).

### Does face typicality influence morph selection?

We modelled each experimental condition (upper and lower face warping) separately, including both *z*-scored perceived and measured typicality as predictors. For each model, we examined the intercept term after an inverse logit transform, which represents the baseline choice of morph for that condition, to which 0.5 (the original image) can be easily compared. Coefficients were exponentiated to the odds-scale. Models were fitted with both informed and uninformed priors as discussed above.

#### Manipulating the lower face (upper face remains constant)

**Fig. 4:**
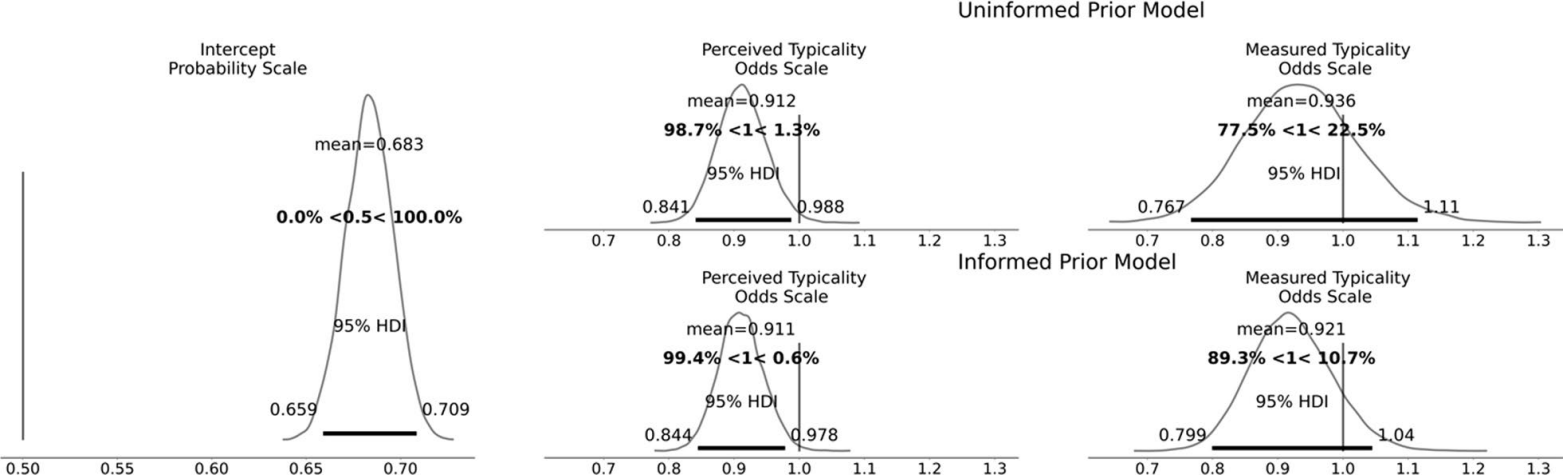
Posterior distribution of coefficients when manipulating the lower face, from models with both informed and uninformed priors. Note the intercept posterior is identical in both models

The posterior distribution of the intercept (after inverse logit transformation) did not include 0.50, *b* = 0.68, 95% HDI [0.66, 0.71], indicating a general tendency to select morph levels in the direction towards the more average shape. For the informed prior model, perceived typicality was associated with lower odds of a more average morph, *b*_odds_ = 0.91, [0.85, 0.98], and the probability that the effect was negative was 99%. For measured typicality, the effect was in the same direction but less certain, *b*_odds_ = 0.92, [0.79, 1.04], and the probability that the effect was negative was 91%. The uninformed prior reduced the effect of perceived typicality only slightly (with the probability of direction being 98%; see Fig. 4), but had more of an effect on measured typicality, with uncertainty increasing and the probability of a negative effect reducing to 76%.

#### Manipulating the upper face (lower face remains constant)

**Fig. 5:**
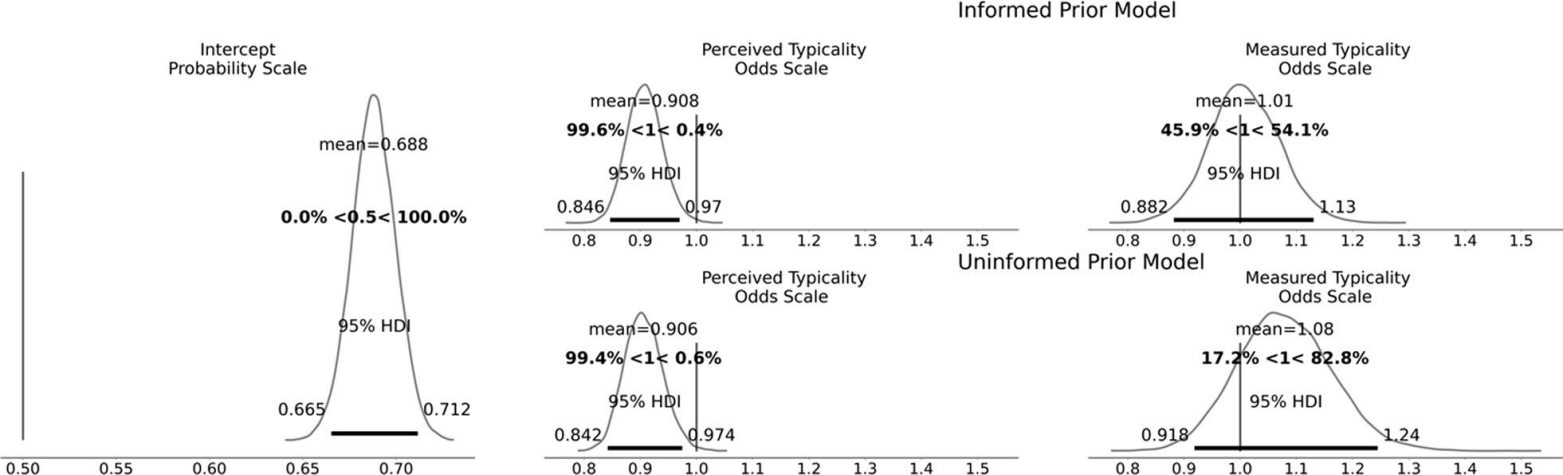
Posterior distribution of coefficients when manipulating the upper face, from models with both informed and uninformed priors. Note the intercept posterior is identical in both models

The intercept posterior did not include 0.50, *b* = 0.69, 95% HDI [0.67, 0.71], indicating again a general trend towards selecting morph levels in the direction of the more average shape. For the informed prior model, perceived typicality was also associated with lower odds of a more average morph, *b*_odds_ = 0.91, [0.85, 0.97], and the probability that the effect was negative was 100%. For measured typicality, however, there was no clear evidence of a negative effect with credible intervals containing substantial mass both above and below one, *b*_odds_ = 1.01, [0.88, 1.11], and the probability that the effect was negative was 46%. The uninformed prior increased the uncertainty of the effect of measured typicality, making a positive effect more likely (with the probability of direction being 17%; see Fig. 5), but had little effect on perceived typicality.

**Fig. 6:**
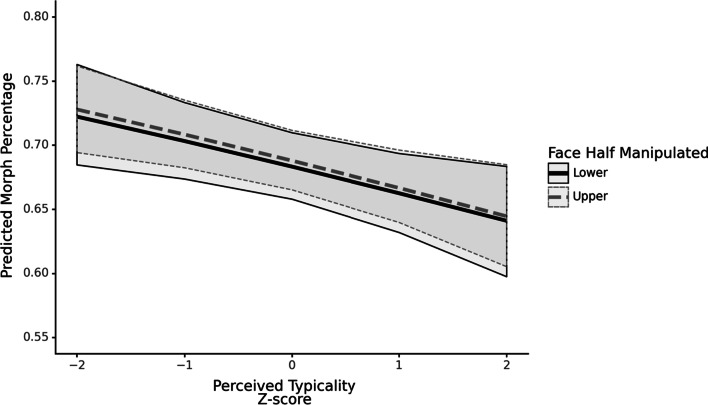
Predictions of both models for varying levels of perceived typicality, holding measured typicality constant. Bands represent the Bayesian 95% credible interval

The above results indicate that greater perceived typicality is associated with a lower choice of morph percentage. Figure 6 visualises the predictions of both models for varying levels of perceived typicality, while holding measured typicality constant, highlighting the range of the observed effect across both conditions. While the predicted morph percentage decreases with increasing perceived typicality, it does not approach 50%, which is the veridical image.

## General discussion

The current experiment aimed to investigate the process through which incomplete faces were completed in terms of the viewer’s internal representation. To this end, we allowed participants to select which version of the original image (morphed along a continuum of shape averageness) depicted how they actually imagined the face to look, having been provided with only half of the original image to base their judgements on. In separate conditions, participants manipulated either the lower or upper half of the face. Our results clearly demonstrated that, in both cases, participants selected versions of these faces that were significantly different from the original images and were made more, rather than less, average in shape.

In addition, we considered the typicality of the original images. In line with previous research (Orghian & Hidalgo, 2020), we predicted that less typical faces (in comparison with more typical ones) would be completed by participants using an even more typical version of the missing part of the face. For faces that were already typical in appearance, there would be a smaller shift towards the average shape when representing the face internally. Indeed, our results supported this prediction, with the original face’s perceived typicality negatively predicting the selected face image’s morph percentage. Importantly, these findings controlled for the measured typicality of the faces. That is, when creating the morphing continuum for each face, the 5% increments between the original image’s shape (0%) and the average shape (100%) differed in the actual amount of change depending on how similar these were to begin with. (A smaller initial difference equated to a smaller change for each increment.) By including this difference within the models themselves (through measured typicality using geometric morphometric techniques), we were able to rule out this potential artefact that could have resulted from the process of stimulus creation.

That incomplete faces were completed using a more average version of the original face provides an underlying mechanism that could explain previous findings where participants rated incomplete faces as more attractive than their original, completed versions, and this was especially true for less attractive faces (Hies & Lewis, 2022; Patel et al., 2020; Pazhoohi & Kingstone, 2022). However, in the current experiment, even the most typical faces were imagined to be more typical when incomplete than they actually were, with the resulting prediction that high-attractiveness faces (synonymous with high-typicality faces) should increase (or at least should not decrease) in rated attractiveness when presented with a face mask. This appears to contradict previous results that saw a detriment to the perceived attractiveness of high-attractive faces when partially obscured (Kamatani et al., 2021; Miyazaki & Kawahara, 2016). Although we did not directly measure the change in attractiveness here, we have discussed earlier (see the Introduction) some of the reasons why there may be disagreement across studies in this field.

It is perhaps worth noting that here, participants were asked to provide ratings of perceived typicality in terms of how much each face deviated from the typical face. Although researchers often rely on the meaning of ‘typical’ as used in everyday language, and so provide no further elaboration (e.g., Dewhurst et al., 2005; Orghian & Hidalgo, 2020), there is evidence to suggest that our measure (deviation from typicality) may show stronger associations with attractiveness than other measures (Morris & Wickham, 2001). Indeed, some researchers have focussed instead on the ease with which a face can be picked out of a crowd (e.g., Hancock et al., 1996; O’Toole et al., 1994), which is thought to place greater emphasis on distinctiveness. As such, future investigations may consider whether this conceptual distinction is important when determining the role of typicality in face completion tasks.

Across two conditions, we investigated how participants responded to incomplete faces when either the lower face or upper face was missing. For both conditions, participants selected versions of the faces that were closer to the average shape. Given that we had no theoretical reason to predict a difference in behaviours, we did not set out to compare responses across these conditions statistically (although the results appear to be similar in terms of pattern). Indeed, we chose to refrain from doing so ‘after the fact’ since the experiment was not sufficiently powered for this comparison. Interestingly, recent evidence suggests that obscuring the lower and upper face may influence attractiveness perceptions differently. Pazhoohi and Kingstone (2022) found that masking the lower face resulted in increased attractiveness for low attractiveness faces only, while masking the upper face resulted in decreased attractiveness for high attractiveness faces only. The authors argued that the eye region was especially important for conveying attractiveness, and so removal of the eyes resulted in a noticeable detriment for highly attractive faces. Again, the current work did not investigate how perceived attractiveness was affected by masking part of the face, and so it remains unclear as to why our findings may not align with those of previous studies.

This experiment utilised only White faces as stimuli, while the majority of our participants also reported being White. Here, we decided to avoid the possibility that our results might be influenced by ethnicity, given that previous research has demonstrated a well-known detriment when responding to ‘other-race’ faces (see Bothwell et al., 1989; Shapiro & Penrod, 1986). If our mental representation of ‘face space’ (Valentine, 1991) is derived from our substantial experiences with faces of our own race then our concept of the average face will also be race-specific (e.g., Jaquet et al., 2008) and presumably less accurate or well-defined for other races. As such, it seems that presenting participants with incomplete faces of other races may lead to interesting effects. For instance, they may be completed using the own-race average shape rather than an average of the race under consideration. Indeed, this idea provides testable predictions that should be investigated in future work, and the same rationale might also be applied to other-age faces (Kuefner et al., 2008). In addition, there is some evidence to suggest an own-gender bias in face recognition (Herlitz & Lovén, 2013). However, it seems unlikely that our mental representation of an other-gender average will be less accurate, given substantial everyday exposure to other-gender faces. However, again, this remains a testable prediction for further study.

The current study demonstrated that participants completed our incomplete faces using versions of the original images that were more average in shape. However, our results raise two interesting questions. First, we chose to create morph continua that increased and decreased the averageness of only the face’s shape to avoid presenting unnaturally smooth skin and unrealistically emphasised blemishes. Intuitively, it seems likely that using a mental representation of the average face to counter missing information would also employ the average texture. That is, I might imagine the lower face under a face mask, for instance, to have average/smooth skin rather than predict specific blemishes or scars based on no prior information. However, with the upper face visible, there may well be reason to generalise the presence of acne, for example, to the imagined lower face. It would therefore be an interesting future direction to try to address this question if suitable techniques were applied to image creation. Second, our morph continua manipulated face averageness and this will likely have altered image attractiveness also. Across identities, our data found that ratings of attractiveness and typicality were strongly associated. It follows, therefore, that increasing the shape averageness of a particular face will have also increased its attractiveness (e.g., Jones & Jaeger, 2019). As a result, we cannot determine whether participants were imagining incomplete faces as (specifically) more average than they really were or (generally) more attractive. Given that research has demonstrated a dissociation between the two characteristics (DeBruine et al., 2007), future studies might attempt to morph faces along both dimensions independently in order to investigate this question.

## Conclusions

In conclusion, when presented with incomplete faces, we have provided the first direct evidence that people complete these images using the average face. Rather than selecting the original, unaltered version, participants chose a significantly more average version as the one they imagined the actual face to look like. Further, this bias towards a more average version was even stronger for faces that were perceived to be less typical. This pattern of results was found when participants were provided with only the lower face or the upper face, utilising both male and female faces. Further research might consider how people approach incomplete other-race and other-age faces, given the hypothesis that our mental representations of the appropriate face averages may be less well developed.

## Supplementary Information

**Additional file 1**. The raw response data provided by participants in each of the two experimental conditions.

## Data Availability

The datasets supporting the conclusions of this article are included within the article and its additional files.
